# SOX2 Is a Potential Novel Marker of Undifferentiated Thyroid Carcinomas

**DOI:** 10.7759/cureus.12102

**Published:** 2020-12-15

**Authors:** Wafaey Gomaa, Azmi Marouf, Asayil Alamoudi, Jaudah Al-Maghrabi

**Affiliations:** 1 Pathology, Minia University, El-Minia, EGY; 2 Medicine, King Abdulaziz University, Jeddah, SAU; 3 Dentistry, King Abdulaziz University, Jeddah, SAU; 4 Pathology, King Abdulaziz University, Jeddah, SAU

**Keywords:** thyroid, carcinoma, tma, sox2, immunohistochemistry

## Abstract

Background

Thyroid cancer is a very common endocrine malignancy. Cancer stem cells are attributable to initiation, progression, and treatment failure in thyroid carcinoma. In the current study, immunostaining of SRY-box 2 (SOX2) in thyroid carcinoma is investigated.

Material and methods

Tissue microarrays were generated from 219 thyroid carcinomas distributed as follows: papillary thyroid carcinoma (175), follicular thyroid carcinoma (11), medullary thyroid carcinoma (11), Hurthle cell carcinoma (three), poorly differentiated thyroid carcinoma (PTDC; nine), and anaplastic thyroid carcinoma (ATC; 10). Immunohistochemistry for SOX2 was done and examined for nuclear staining. The results were analysed.

Results

SOX2 immunostaining was positive in one PDTC (out of nine; 11.1%) and in three ATC (out of 10; 30%). The rest of the thyroid cancers showed no immunostaining for SOX2.

Conclusion

The study represents for the first time SOX2 immunostaining on a large number of thyroid carcinomas. We discovered that SOX2 immunostaining is found in PDTC and ATC while SOX2 immunostaining is lacking in other thyroid cancer. SOX2 may be a marker of loss of differentiation in thyroid carcinoma. In vitro as well as in vivo molecular studies are required to explore the possible role of SOX2 in thyroid carcinoma.

## Introduction

Thyroid cancer is the most common malignancy of endocrine organs. Its incidence continues to rise worldwide [1,2]. Thyroid cancer is one of the most prevalent cancers in Saudi Arabia. In 2014, it was the second most common female cancer in incidence (11.5% of all cancers) and represented 4.2% of all cancers in males for that year [3]. The major subtypes of thyroid cancer include papillary thyroid carcinoma (PTC), follicular thyroid carcinoma (FTC), medullary thyroid carcinoma (MTC), poorly differentiated thyroid carcinoma (PDTC), and anaplastic thyroid carcinoma (ATC) [4]. Resistance to chemotherapy is problematic in treatment of thyroid carcinoma. This resistance is related to failure of current medications to target cancer stem cells (CSCs) [5,6].

SRY-box 2 (SOX2) is a transcription factor that is important for tissue regeneration [7] and is well recognised to have critical roles in human and other mammalian embryogenesis [8]. In cancer cells, SOX2 is involved in regulation of cellular proliferation, signalling in apoptosis, invasion, and migration [9]. SOX2 has been detected in different cancer types [10]. A lower SOX2 expression in CSCs promotes greater cell death and response to cisplatin and doxorubicin [11]. SOX2 is expressed in thyroid CSCs [12] and may be an important marker in thyroid CSCs [11].

In the current study, the aim is to investigate the incidence of immunostaining status of SOX2 in thyroid carcinomas and to find the possible relation to clinicopathological features of tumours.

## Materials and methods

Patients

The study involved paraffin wax tumour blocks from 219 patients diagnosed with thyroid cancer. The blocks were obtained from the archives of the Department of Pathology at King Abdulaziz University, Jeddah, Saudi Arabia. Some clinicopathological characteristics of patients are listed in Table 1 and Table 2. The study was performed after obtaining agreement of the ethics committee of Faculty of Medicine, King Abdulaziz University, Saudi Arabia (IRB #1127-13). The study was done in accordance with the Declaration of Helsinki.

**Table 1:** Clinicopathological features of tumours

Feature	Category	n (%)
Gender	Female	167 (76%)
	Male	52 (24%)
Age (Range 13-86)	< 45 years	125 (57%)
	≥ 45 years	94 (43%)
Extrathyroid Extension	Absent	182 (83%)
	Present	37 (17%)
Focality	Unifocal	130 (59.4%)
	Multifocal	89 (40.6%)
Lymphovascular Invasion	Absent	178 (81.3%)
	Present	41 (18.7%)
Capsular Invasion	Absent	176 (80.4%)
	Present	43 (19.6%)
Nodal Metastasis	Absent	30 (13.7%)
	Present	54 (24.7%)
	Not assessed	135 (61.6%)
Surgical Resection Margin	Free	159 (72.6%)
	Involved	60 (27.4%)

**Table 2:** Histological subtyping of thyroid neoplasms included in the study

	Number (%)
Papillary Thyroid Carcinoma (PTC)	175 (80%)
Follicular Thyroid Carcinoma (FTC)	11 (5%)
Medullary Thyroid Carcinoma (MTC)	11 (5%)
Hurthle Cell Carcinoma (HCC)	3 (1.4%)
Poorly Differentiated Thyroid Carcinoma (PTDC)	9 (4.1%)
Anaplastic Thyroid Carcinoma (ATC)	10 (4.5%)
Total	219 (100%)

Tissue microarray

Tissue microarrays were designed and generated from thyroid cancers. A tissue microarrayer (the automated TMA Master 1.14 SP3 [3D Histech Ltd., Budapest, Hungary]) was used. Placenta tissue was used for orientation. TMA blocks were cut with a thickness of 4 µm and kept on positive-charged slides (Leica Microsystems Plus Slides; Leica GmbH, Wetzlar, Germany).

Immunohistochemistry

Sections were prepared for immunostaining by deparaffinisation in xylene and rehydration. Immunostaining was performed on BenchMark XT, Ventana® automated immunostainer (Medical Systems Inc., Tucson, AZ, USA). Pre-treatment was done using CC1 (prediluted cell conditioning solution) for 60 min. Monoclonal rabbit anti-human SOX2 antibody (SP76; Cell Marque Corp., Rocklin, CA, USA). A DAB detection kit was used (Ventana® I-view). The slides were washed as appropriate, counterstained with Mayer’s haematoxylin and mounted. Negative control and positive control slides were included. SOX2 nuclear immunostaining was reported as the percentage of positive cells.

Statistical analysis

Descriptive statistics were performed using Statistical Package for Social Sciences (SPSS) version 16 (IBM Corp., Armonk, NY, USA).

## Results

**Figure 1:**
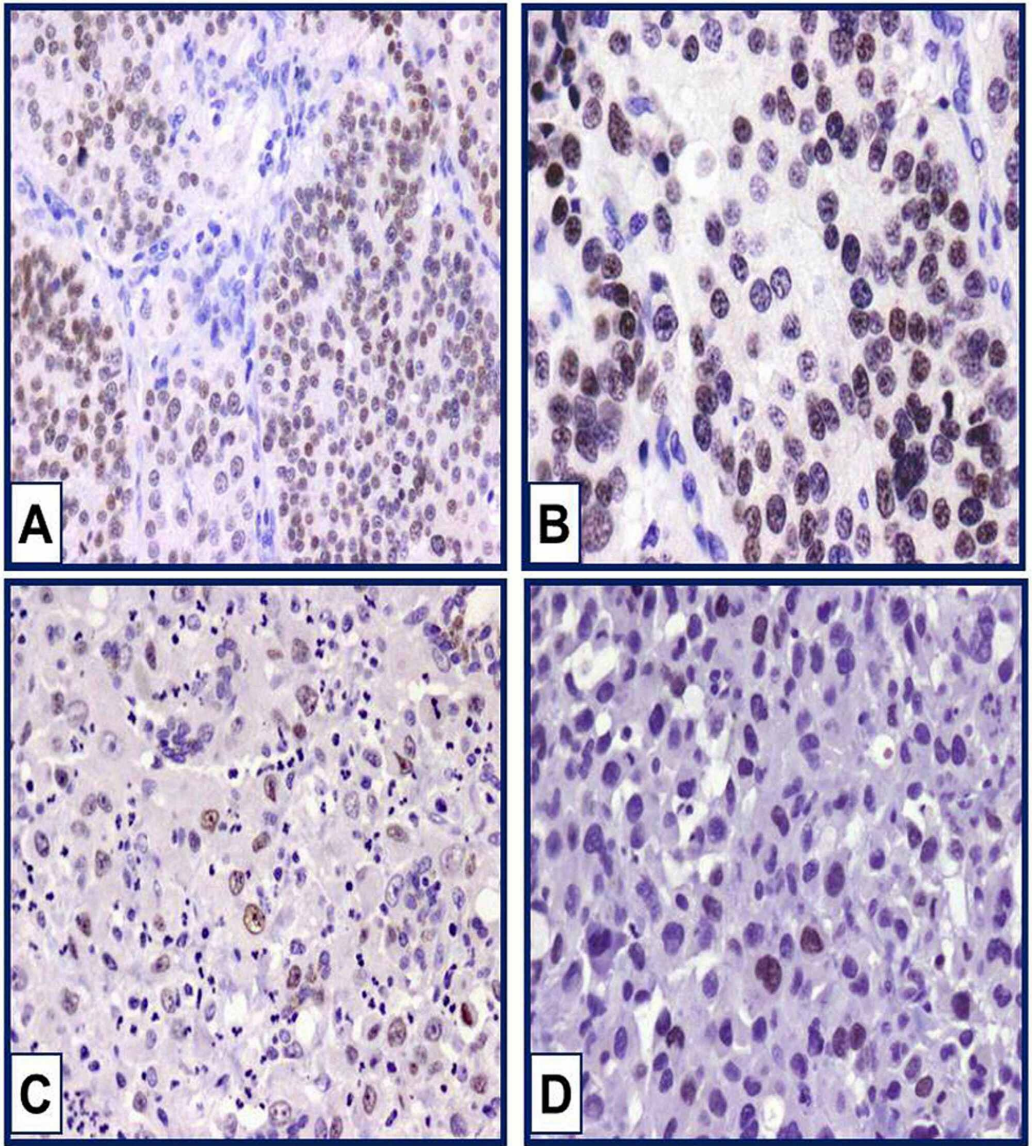
SOX2 immunostaining in PDTC and ATC Moderate nuclear immunostaining of SOX2 is noticed in poorly differentiated thyroid carcinoma (A). Original magnification was 100x. Weak and moderate nuclear immunostaining of SOX2 in anaplastic thyroid carcinoma (B, C, and D). Original magnification was 200x. PTDC: poorly differentiated thyroid carcinoma; ATC: anaplastic thyroid carcinoma

In the present study, we demonstrated nuclear SOX2 immunostaining in some thyroid carcinomas. Positive nuclear immunostaining was observed only in PDTC and ATC. SOX2 immunostaining in other types of thyroid carcinoma was not detected. Nuclear immunostaining was moderate in intensity and diffuse in distribution. SOX2 immunostaining was observed in one out of nine PDTC (11.1%), and positive in three ATC out of 10 (30%). The percentage of SOX2 immunostaining in PDTC was 35%. The positive PDTC belongs to an elderly male with nodal metastasis, capsular invasion, positive margin, positive vascular invasion, and a tumour size of 5.5 cm. In ATC, the mean percentage of positivity was 40%. SOX2-positive ATC patients were females. SOX2-positive ATCs were associated with nodal metastasis, capsular invasion, extrathyroid extension, vascular invasion, positive margins, and large-sized tumours. In addition, distant metastasis was detected in one SOX2-positive ATC. Figure 1 shows positive nuclear immunostaining in PDTC and ATC.

## Discussion

SOX2 is a transcription factor belonging to the high-mobility SRY-related HMG-box (SOX) family. It plays a vital role in determination of cell fate and maintaining undifferentiated embryonic stem cells, thereby regulating developmental processes [7,13,14].

Abnormal SOX2 expression has been found in different cancers and is correlated with the presence of CSCs [15-17]. In most types of cancers, SOX2 acts as an oncogenic transcriptional factor and is elevated in malignant tissue as compared to normal tissue [8,18-23]. Also, downregulation of SOX2 was found to suppress growth and metastasis of lung cancer [19]. On the other hand, in some studies SOX2 is thought to have a tumour suppressing action based on its reduced expression in some types of cancer, and overexpression inhibited cell proliferation and resulted in cell-cycle arrest and apoptosis [24]. In normal urothelial cells, SOX2 expression was not detected while in pre-neoplastic bladder lesions SOX2 was seen and continued to be expressed in invasive neoplastic lesions [25].

In the current study, we investigate the pattern of SOX2 immunostaining in a large number of thyroid carcinomas for the first time. In thyroid cancer cells, SOX transcription factors were detected in thyroid cancer cell lines [26]. Silencing SOX2 expression in ATC cell lines was associated with a decrease in stemness genes (Nanog and Oct4) expression and increased the sensitivity to chemotherapeutic agent-induced cell death [11].

The current study reported for the first time SOX2 immunostaining on tissue microarrays done from a large number of thyroid carcinomas. In the present study, all PTC were negative for SOX2 immunostaining. This finding is supported on PTC cell lines where SOX2 was reported as a marker of loss of differentiation [27]. In our study, SOX2 immunostaining was detected in 11.1% of PDTC. In a previous in vitro study, PDTC cell lines showed that SOX2 was not expressed [28]. So far, we reported for the first time immunoexpression of SOX2 in PDTC. Although we have a small number of cases, this finding is worthy to be checked especially when all PTC are negative for SOX2. The second interesting finding in our study is SOX2 positivity in 30% of cases of ATC. ATC is a rare, highly undifferentiated neoplasm with aggressive and morbid clinical outcome. Surgery is usually palliative, and radiotherapy and chemotherapy are not fully effective. This may be related to inadequate targeting of the cancer-initiating cells [29]. SOX2 appears to play a pivotal role in the resistance of ATC cell lines to chemotherapy through maintaining cell self-renewal by involvement in cell signalling and protein-protein interactions. SOX2 variable positivity in ATC was shown in one study in four out of eight specimens and in an ATC cell line [11]. Our finding is in agreement with this study where the average percentage was around 35%. This is supporting the role of SOX2 in ATC.

We suppose that SOX2 can be used in diagnostic work as a novel marker for undifferentiated thyroid cancers. The limitation of this study is the low number of PDTCs and ATCs and the absence of benign and normal tissues.

## Conclusions

We hereby present for the first time SOX2 immunostaining on the largest cohort of thyroid carcinomas. Interestingly, SOX2 immunostaining was confined to thyroid carcinomas with lack of differentiation (PDTC and ATC). SOX2 is a potential marker of these subtypes of thyroid carcinomas and may help in identifying metastatic ATC and PDTC. These findings, albeit found in a small number of cases, are still interesting and need more confirmation on a larger number of ATC and PDTC. In addition, the prognostic implication of SOX2 immunostaining in these tumours need to be addressed.
